# “I'm a bathroom expert”: a qualitative study exploring how students with physical disabilities manage toileting during college

**DOI:** 10.3389/fped.2024.1397229

**Published:** 2024-09-27

**Authors:** Oluwaferanmi O. Okanlami, Jodi M. Kreschmer, Saumya Gupta, Allison Lee, Aruna V. Sarma, Courtney S. Streur

**Affiliations:** ^1^ Department of Family Medicine, Michigan Medicine, University of Michigan, Ann Arbor, MI, United States; ^2^ Department of Physical Medicine and Rehabilitation, Michigan Medicine, University of Michigan, Ann Arbor, MI, United States; ^3^ Department of Urology, Michigan Medicine, University of Michigan, Ann Arbor, MI, United States; ^4^ Division of Student Life, Michigan Medicine, University of Michigan, Ann Arbor, MI, United States; ^5^ Department of Urology, University of Michigan, Ann Arbor, MI, United States; ^6^ Department of Epidemiology, School of Public Health, University of Michigan, Ann Arbor, MI, United States; ^7^ Department of Pediatrics, Michigan Medicine, University of Michigan, Ann Arbor, MI, United States

**Keywords:** spina bifida, cerebral palsy, disability, persons with disabilities, neurogenic bladder, neurogenic bowel

## Abstract

**Introduction:**

Health care providers caring for youth with physical disabilities encourage them to be as independent as possible, which includes obtaining higher education when feasible. However, little is known about the experiences of higher education students in managing their toileting.

**Methods:**

We performed 1:1 semi-structured interviews with 13 current college students with physical disabilities (4 male, 9 female), of whom six were on a formal bladder and/or bowel management program. Three researchers analyzed all transcripts using constructivist grounded theory procedures.

**Results:**

We identified six themes, including: (1) adherence to prescribed programs, (2) importance of time management, (3) interfering with class, (4) balancing intake and toileting, (5) campus bathroom experiences, and (6) acclimating to new living situations. Students needed strong personal skills in time management, adaptability, and self-advocacy to both manage their toileting needs and engage in academic and social activities. This often took time to develop while in college. They faced barriers such as a lack of private, well-maintained bathrooms.

**Conclusions:**

Health care providers should encourage their patients to develop these personal skills prior to starting college, while colleges need to better support these students through honoring their accommodation needs and ensuring the availability of private, accessible bathrooms.

## Introduction

A primary goal of health care providers for emerging adults with physical disabilities is promoting their maximal independence. Independence, in turn, enables them to enjoy mature relationships and strive for professional and personal goals. When feasible, achieving maximal independence often includes pursuing a degree in higher education, whether a certificate from a community college, a bachelor's degree from a college, or a graduate degree from a university. Health care providers play a critical role in supporting these emerging adults interested in pursuing higher education. Specifically, urologists as well as physiatrists, neuropsychologists, and occupational and physical therapists often support these emerging adults in both achieving their desired level of continence and learning to manage their toileting as independently as possible.

However, the ability to support these emerging adults is limited by the current lack of understanding of the lived experiences of students with physical disabilities in managing their bladder and bowels in the college or university setting. Depending on the disability type, some students may be on a formal bladder program, such as intermittent catheterization per urethra or a surgically constructed bladder channel, medications, timed voiding, or a combination of these. For these students, their bladder programs are crucial not only to achieve continence, but also to avoid urinary tract infections, maintain kidney health, and avoid potential morbidity such as bladder rupture in those who have undergone bladder augmentation. Other students may be on a formal bowel management program, which may include timed defecation, medications, or small or large volume enemas which may take an hour to complete each day. Some students may need assistance with toileting and, whether on a formal program or not, all need to learn to adapt to new bathroom facilities. Many young people experience a decline in adherence to toileting programs as well as a decline in bladder and bowel health in early adulthood (1–7). However, it is not clear if this is also true for people in higher education, how young people balance their toileting needs with their academic load and social involvement, and what barriers or facilitators they may face.

In this context, we performed an exploratory qualitative study of students with physical disabilities in community college, undergraduate, or graduate school programs to learn about their experiences with managing their toileting while in higher education. This was a part of a larger study investigating the experiences of these young people during college and opportunities to better support them.

## Methods

Institutional Review Board approval was obtained prior to participant recruitment (HUM0022833).

### Research team

The research team was composed of five members: a physician and director of several disability services and adaptive sports and fitness for a large public university (OOO), a research manager within the school of medicine (JK), a project coordinator within the university (SG), an undergraduate student (AL), an epidemiologist (AVS), and a pediatric urologist (CS). OOO, JK, SG, and CS have occupational or research expertise in the lived experiences of young people with disabilities. JK and CS have expertise in qualitative research methods and the other team members were trained in these methods for this study. The research team included members with a physical disability who engaged in all steps of the research study.

### Study sample

Current community college, undergraduate, or graduate students ages 18 years or older with a physical disability (defined as a condition impacting mobility, such as cerebral palsy, spina bifida, spinal cord injury, or arthrogryposis) were included. To capture the range of experiences of students with physical disabilities, students who self-identified as having a physical disability based on this definition were eligible, whether or not they used a mobility aid. Students were excluded if they were not currently enrolled in higher education, if they did not have a condition associated with a mobility impairment, or if they were not fluent in the English language.

### Recruitment

Recruitment was conducted using convenience and snowball sampling between February 1st and April 30th 2023. Advertising via social media was conducted by posting information about the study on local and national disability organizations’ social media accounts as well as the account of one of the study's authors (OOO). A letter and flyer describing the study were sent to disability offices at several universities. All advertisements provided a link to an informational page on our institution's research outreach website, which further described the study and shared the contact information for the Research Manager. One participant was recruited from CS's clinic. Finally, participants were asked at the end of their interviews if they knew of others who may be interested in participating.

Those interested in the study contacted the Research Manager (JK) who further explained the study, screened for eligibility and set up a time for the interview. Comprehensive written or phone consent was obtained, with a copy of the consent form emailed to the participant. Of 14 of students who expressed interest, 13 completed screening and 13 participated with one person not following-up after expressing interest.

### Qualitative data collection

Three members of the research team (OOO, JK, and CS) developed the semi-structured interview guide based on their expertise in supporting higher education students with disabilities (OOO) and clinical and research expertise in the experiences of young people with disabilities (JK and CS). The guide was reviewed with the remainder of the research team and employees from a large university's disability office. The interview guide was revised based on feedback. Additionally, the interview guide was iteratively modified based on new information learned during the study (Supplementary Material). This was a part of a larger study probing the broader experiences of young people with physical disabilities in higher education.

One-on-one semi-structured interviews were performed via the institution's secure Zoom account. All interviews were conducted by the Research Manager (JK), recorded, transcribed verbatim, and de-identified. Each participant was assigned a study identifier to ensure anonymity.

### Data analysis

Four authors (JK, SG, AL, CS) used constructivist grounded theory procedures to analyze the interviews (8). This inductive approach was chosen as this is an area with little foundational knowledge.

NVivo (QRS International) was used to document the coding. The coders reviewed each transcript in its entirety independently, then together reviewed the transcripts line by line. Quotes that answered the research questions in a meaningful way were highlighted and assigned a short descriptive phrase or code reflective of the meaning of the quote. Focused coding was then conducted to synthesize the most common and significant codes and identify their dimensions (9). The code book was iteratively refined by the coding team during ongoing analysis to combine overlapping codes and to ensure that the meaning of the codes was clear. Abductive logic was employed to identify theoretical explanations for the findings whereby a theory was proposed based on the data and influenced by the backgrounds of the coders and then evaluated and further refined with additional interview data (10). The coders discussed all codes and theories together until consensus was reached, with discrepancies being reviewed with an additional member of the research team (OOO). After 13 interviews, thematic saturation regarding overall higher education experiences was reached, meaning all dimensions and a thorough understanding of each theory or theme was fully understood and supported by the data (11). Next, those themes specific to toileting were re-reviewed to confirm thematic saturation relevant to the question of managing the bladder and bowels while in higher education. Reaching thematic saturation, the occupational, research, and lived experiences of the research team, rigorous discussion of the findings, and the ability to achieve consensus contributed to the trustworthiness of the data (12, 13). Member checking and repeat interviews were not performed.

## Results

### Participants

Thirteen higher education students participated in the study (Table 1). Of these, 9 were female and 4 male, the majority had cerebral palsy (*n* = 6), while the remainder had spinal cord injury (*n* = 3), spina bifida, traumatic brain injury, Ehlers Danlos Syndrome, or complex regional pain syndrome (the later each *n* = 1). Four participants were on a formal bladder and bowel program and two were on a bowel program only. The experiences of these six participants in managing their formal programs as well as the broader group in managing their toileting are reported here.

**Table 1:** Demographic and college data of participants.

#^a^	Age	Gender	Disability type	Ambulatory aids	Bladder management	Bowel management	Type of school
1	25–29	Male	Cerebral palsy	Braces/splints	None	None	Graduate (private)
2	20–24	Female	Cerebral palsy	None	None	None	4 year undergraduate (private)
3	20–24	Female	Cerebral palsy	Wheelchair	Timed voiding with physical assistance	Physical assistance	Community College (public)
4	25–29	Female	Cerebral palsy	Walker	None	None	Graduate (public)
5	15–19	Female	Spinal cord injury	Wheelchair	None	Enemas	4 year undergraduate (public)
6	20–24	Male	Traumatic brain injury	None	None	Suppositories	4 year undergraduate (public)
7	15–19	Female	Cerebral palsy	Braces/splints	None	None	4 year Undergraduate (public)
8	20–24	Female	Spina bifida	Wheelchair	Intermittent catheterization	Enemas	Graduate (public)
9	20–24	Male	Cerebral palsy	Walker	None	None	4 year undergraduate (public)
10	20–24	Female	Ehlers Danlos syndrome, hearing disability	Braces/splints	None	None	4 year undergraduate (private)
11	15–19	Female	Complex regional pain syndrome	Scooter	None	None	4 year undergraduate (private)
12	15–19	Female	Spinal cord injury	Wheelchair	Intermittent catheterization	Enemas	4 year undergraduate (private)
13	25–29	Male	Spinal cord injury	Wheelchair	Intermittent catheterization	Suppositories	4 year undergraduate (public)

^a^ Participant number.

Six themes emerged regarding the participant's experiences with toileting while in higher education: (1) adherence to prescribed programs, (2) importance of time management, (3) interfering with class, (4) balancing intake and toileting, (5) campus bathroom experiences, and (6) acclimating to new living situations.

### Adherence to prescribed programs

Students varied on their reported adherence to programs, with some following it less closely and others becoming more vigilant. Those who followed their programs less closely in college reported their class and social schedules were barriers that interfered with their programs.

“I think [I am] probably less [strict with my bladder program]. I mean, obviously like I still do it when I'm supposed to. But obviously like sometimes I have to change my schedule, so that's probably not the best, but it works, so it's fine.” -Participant 5

Those who reported becoming more adherent to their schedules were motivated to do so to avoid illness or because they felt more responsible for their health and health management now that they lived independently.

“[I am] probably more strict [with my bladder and bowel program] because I don't want to take any sick days… when I was. younger I would not catheterize as much…I hated doing enemas… I would have appointments… and they would always tell me you need to…catheterize this amount of times… But I never really paid attention until…I moved out of home. Like, oh yeah, I need to look after myself.” -Participant 8

### Importance of time management

Students discussed how critical it was to learn time management when they started college to balance their toileting needs with their school and extracurricular schedule. For some, this involved learning a new skill, while for others this involved honing their existing time management skills to adjust to their greater independence and responsibility. For one student, this required not only determining his scheduling needs for his own self, but also for a nurse who helps with his toileting.

“I have a nurse that comes, and they help me with [cathing]. It's just very important that you know your schedule because they don't. It's not their responsibility to know your schedule. You have to, as a college student, get your itinerary down, so that way you can be successful here at school.” -Participant 13

Others noted learning good time management helped them avoid missing class due to toileting needs, especially given the increased time toileting may take.

“I catheterize and do enemas. For me, it's just getting in a rhythm… Like I have got a rhythm or system where, oh, I need to make sure I pee before I’m going to class because it's normally an hour and a half or maybe two hours. So it's just kind of getting into those rhythm and system because I don't want to end up like having to go out of class and then miss some of it and then probably taking, I don't know, ten minutes to go to the toilet or stuff like that.” -Participant 8

Finally, several students noted that the bowel program took the greatest amount of time and was most important to schedule intentionally. One student reported looking ahead at her schedule to identify and plan the best time for the bowel program.

“I guess like with my bowel program, I just have to schedule it, depending on my class work or test or exams, just to plan out when I should do it…sometimes I have to do it earlier or later.” -Participant 5

Conversely, another found a time she could do it consistently, which she found freed her to participate in college life more fully.

“I think the important thing is just like figuring out what time works best for you, especially for a bowel program. And for me, personally, I find that in the morning works best for me because then I can just get it all out of the way, and the rest of the day I don't need to worry about it. And, you know, that gives me more time in the evening to either focus on homework or hang out with friends or engage in other social activities.” -Participant 12

However, she learned it was important to build in buffer time.

“Again, it's just time management…making sure you consider how much time it can take because… one day it only takes 30 min, and then another day it takes a full hour…So give yourself like ample time in the bathroom, whether that means… waking up a bit earlier…” -Participant 12

### Interfering with class

Despite proactive planning, toileting needs interfered with class on a regular or episodic basis for students. Several students, including those not on formal bladder or bowel programs, mentioned the need to use the bathroom urgently and, for some, frequently. This was considered unacceptable by some professors.

“…I need to pee like frequently and very quickly once I realize it…. it's definitely something where I need to talk about, with professors. Because even if they say like just go to the bathroom whenever, I have found that they sometimes will comment because it might be like twice in a class period…” -Participant 10

One student took courses virtually in part due to her need for bathroom assistance and her fecal urgency.

“…for me…especially the bowels, it's something that comes on, and it's like now. Like I don't get a lot of warning… And even if I were to go [to in-person classes instead of Zoom], and I need some like assistance going to the bathroom, so I don't know that there are people at the college that they're trained and would help with that.” -Participant 3

For others, occasional accidents caused them to miss class.

“I've experienced one accident before, and that was like right before my class and was able to clean myself up and get back into my wheelchair. However, I knew that…with a bowel accident, to manage that, I didn't have the supplies on me. I needed to go back to my dorm.”—Participant 12

### Balancing intake and toileting

Whether on a formal program or not, students described carefully considering how much they drank and what they ate as this determined their need to use the bathroom. Several noted the importance of drinking water and regularly using the bathroom to keep themselves healthy.

“I think [my urinary tract infections were] a lot intertwined with like constipation and stuff too. And a lot of it was not enough water and not going to the bathroom as much.” -Participant 3

However, water intake needed to be balanced with bathroom use. A healthy diet was also considered important to avoid constipation.

“I mean, no, if you can't take yourself to the bathroom… Like you don't want to chug water and stuff. But for like the constipation too, like adequate amounts of water and like the way you eat and stuff.” -Participant 3

### Campus bathroom experiences

Relatedly, most students had to think ahead about where they may use the bathroom during the day. They had negative perceptions of many bathrooms around campus. Often, the accessible bathrooms were broken or used by people without disabilities. One participant described their system for staying up to date on the availability of accessible bathrooms.

“…with like the disability groups that do exist on campus…messaging group chats, hey, this toilet is out…” Participant 10

Additionally, students described many bathrooms as insufficiently private.

“in the U.S., the toilets are very weird like in terms of their disabled toilets. And you [can] see like above and below, and you can kind of sometimes see in the gaps, which I find really weird… it makes it less private… you've got to catheterize and those type of things, it's kind of awkward.” -Participant 8

Through experience, students learned where the closest acceptable bathroom was located around campus at all times and what bathrooms to avoid.

“in the library… all of the stalls, they have shower curtains for doors instead of doors, which, it was like I just walked in the bathroom, and I left… in some of the bathrooms, they have kind of absurdly high, the stalls… yeah, some of the bathrooms here are truly horrendous. And I'm a bathroom expert, I think I would consider myself.” -Participant 6

### Acclimating to new living situations

Students described that it took time to figure out the best way to manage their toileting in college.

“…it's true for any…new environment that you go in, whether it's on vacation or…you move to a new house or whatever. You have to learn how to work with your body in this new environment. And then with time and practice, everything will get quicker.” -Participant 12

One participant described learning to be completely independent in performing her bowel management and responsible for her medical supplies in college.

“…for my bowel program, I have to transfer onto a chair. And before I came to college, I wouldn't really do that independently… But it definitely like made me have to learn how to do it…And just like the whole process of doing the bowel program, I had to learn how to do it, just setting everything up and being proactive about having all the supplies and knowing how much I have…I actually had surgery where I can now cath out of my belly button…it's pretty cool. I can totally do that independently now…” -Participant 5

Supplies took up a lot of space in college student's small housing.

“…I use the cath, so they're in big boxes, so that's been a big part of storage space that I've needed…there's huge boxes in my closet…” -Participant 5

The bathroom setup was an especially important consideration for students in choosing or dealing with their student housing. Some managed to get a private bathroom, which was considered by most to be ideal but more expensive than regular dorms.

“So I live in a premium housing dorm… premium housing costs a tad bit more money than regular housing… it's worth it because I'm living by myself… And I get my own bathroom, so I don't have to share a bathroom with anybody.” -Participant 2

Others, however, who tried to secure a private bathroom were unsuccessful.

“I was supposed to have a bathroom, but they didn't give me a bathroom, which is really annoying because I also have ulcerative colitis, so I have to go to the bathroom like 12, 13 times a day.” -Participant 6

Those who had to share a bathroom, whether in a suite situation or with a common bathroom for the entire floor, had to learn how to find private time in the bathroom. This was especially important for those on a bowel program. One participant described dealing with this proactively by creating a bathroom schedule with her roommate. She found this helpful for establishing expectations and maintaining a positive relationship.

“For me, specifically, I need a good chunk of time in the bathroom in the morning, and so [I asked my roommate] to be flexible with me and work with me in terms of like just sharing the bathroom and who's getting the bathroom at what time.” -Participant 12

## Discussion

**Figure 1:**
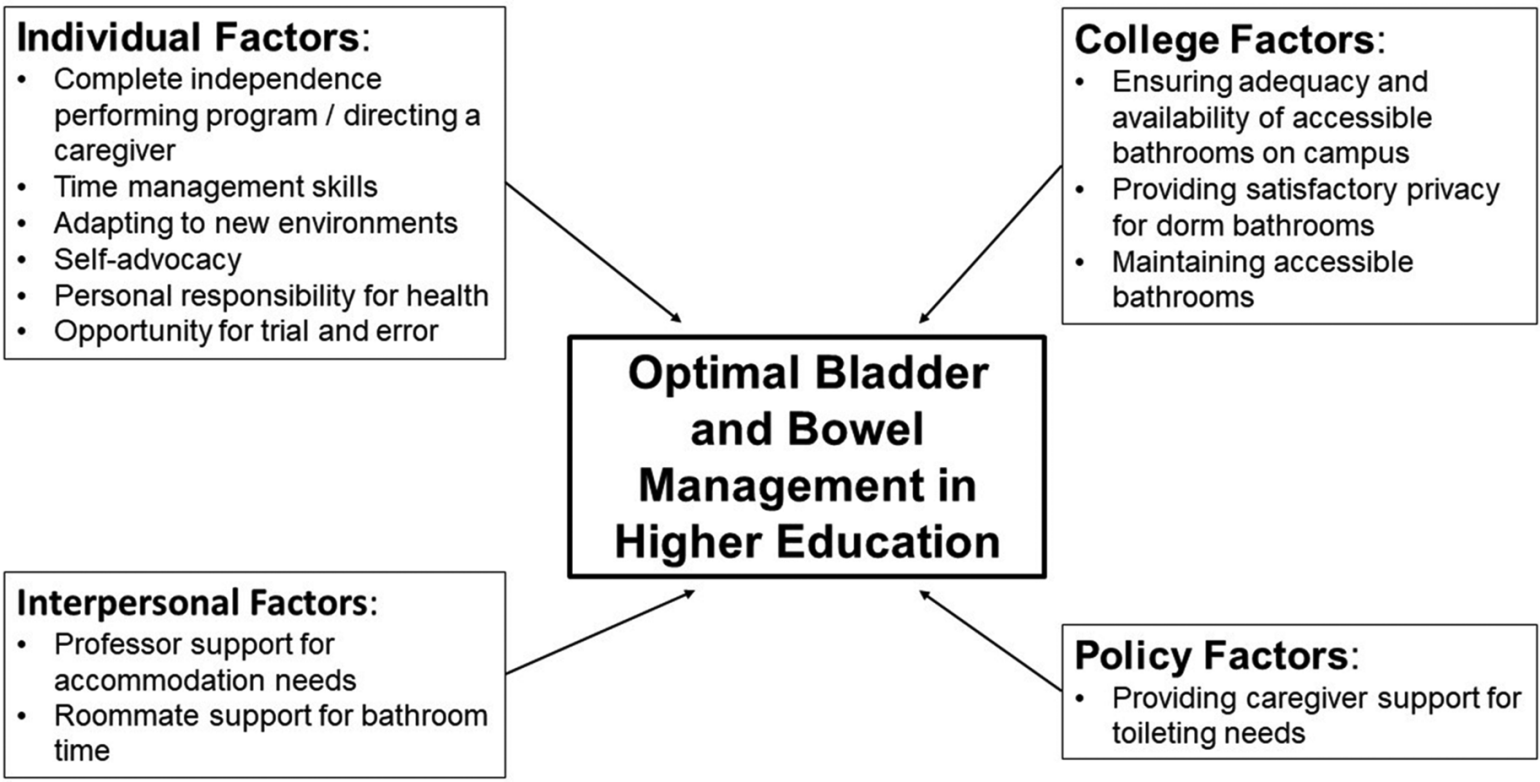
Socio-ecological factors facilitating optimal bowel and bladder management in higher education.

Toileting needs impacted the college experiences of students with physical disabilities in this study. The barriers and facilitators to managing toileting needs in college can be understood using a socio-ecological framework, which recognizes the multilevel influential factors (Figure 1). On the individual level, developing strong personal self-management (e.g., planning ahead and time management) and self-advocacy (e.g., speaking up for ones needs with roommates and professors) skills were critical facilitators for success. College was often a period of great personal growth in these areas. Students described both becoming more independent in self-care, but also feeling more responsible for their health. Interpersonal experiences with roommates and professors served as both barriers and facilitators. Although the students in this study reported positive interactions with roommates, they had mixed interactions with professors, with some being understanding of their accommodation needs (e.g., being allowed to leave the classroom as needed) and others not. The college's commitment to supporting these students by providing readily available, private, well-maintained accessible bathrooms around campus was a key barrier identified by most students in this study. The lack of appropriate bathrooms impacted students’ daily lives and even their choices regarding their hydration. Finally, current policy is another critical barrier to these students. While policy supports care providers such as school nurses and Para pros for students prior to college, the same level of support is not available to college students. As a result, one student resorted to virtual classroom participation, missing the benefits and potential personal growth of an in-person experience. Additionally, appropriate student housing with features such as fully accessible and available bathrooms were not readily equitably available and required students to pay more. Strikingly, together this demonstrates that managing toileting needs in the college setting relies on the personal skills of the person, with some professors, colleges, and policy serving as barriers to their success.

The themes identified in this study align with the existing literature in several ways. Adherence to a bladder and bowel program can be challenging for all people, with a decline for many around the time of achieving independence (14). These students reported an adjustment period of learning both the importance of and how to manage a program independently. Other studies have demonstrated that a lack of coverage for caregiver support for those on bladder and bowel programs negatively impacts adherence (15, 16). In this study, the lack of support did not impact adherence, but did prevent one student from the opportunity to participate in in-person classes. These students found their toileting often interfered with class. Likewise, other studies have also reported that bowel and bladder needs can more generally interfere with efforts to be active outside the home (17–19). Similar to student's report of campus bathrooms, other studies have reported that public bathrooms often do not have enough or adequate accessible stalls, are not well maintained, do not considerer factors that could support people with disabilities (e.g., a table to lay out supplies), and are often used by people without disabilities (19, 20). As students in this study had to intentionally plan their intake and day around their schedule and the availability of bathrooms, those in other studies have reported that the lack of reliable public bathrooms and support for toileting assistance is a source of anxiety (20). While not reported by students in this study, others have demonstrated that this anxiety cause some to miss social activities (20).

This study adds to the existing literature in several ways. First, it demonstrates that managing toileting in higher education is a challenge for many students with physical disabilities, regardless of if they are on a formal bladder or bowel program. This is due to both to bathroom availability, accessibility, and privacy concerns as well as several students describing bladder and bowel symptoms (e.g., frequency, urgency) despite not being on a formal bladder or bowel program. This could be related to the emergence of bladder and bowel symptoms in early adulthood for people with certain conditions such as cerebral palsy, which may be further exacerbated by difficulties with bathroom accessibility (21–24).

Second, based on the results of this study as well as our personal, clinical, and occupational expertise and the existing literature, we have developed a conceptual model of maximizing bladder and bowel management in higher education. The students in this study identified six factors of individual growth that allowed them to be successful in managing their bladder and bowel program while in college (Figure 1). These included (1) opportunities for trial and error for learning, (2) complete independence in performing a program or directing a caregiver, (3) ability to adapt a program to new environments, (4) proactive time management skills, (5) self-advocacy, and (6) personal responsibility for health and health-related habits. Medical providers and families of aspiring higher education students with physical disabilities can help prepare adolescents by targeting opportunity and growth in each area. For example, the adolescent can be encouraged to figure out for themselves how to adapt their program when they are on vacation or at a new place. Families and medical providers can work together to help the adolescent become completely independent with performing a bladder and bowel program. Adolescents can be prompted to learn to review their class, studying, and social schedule and determine when they should perform their program. They could also be made responsible for communicating their accommodation needs to teachers. Certainly all of these may be made more challenging and require longer time to develop if the adolescent also has executive functioning limitations, which is common in adolescents with certain congenital disabilities. The timing of this development may vary by adolescent (14, 25–27).

Allowing for trial and error may be most challenging for families and medical providers. Although this is an important part of learning and growth, families and medical providers may be fearful of the implications of an “error.” (28–31) Many families have maintained a high level of control to take excellent care of their child, which may be challenging to gradually shift (32, 33). Nonetheless, providers and families should think of ways to allow for this gradually allow for this while the adolescent is still at home. For example, families could start by allowing them to control their fluid intake and help them understand the outcomes, both positive and negative.

However, the personal efforts of the aspiring students must be met with better support from professors, colleges, and health policy. Ideally, students should meet with their campus's Disability Office prior to starting classes. The Disability Office drafts a letter explaining the accommodation needs of the students, without disclosing any diagnosis or explanation of the need. Professors should make these students feel well supported, ensuring their accommodation needs are met and that the students feel comfortable using them without question, such as leaving the class as needed. There is an urgent need for colleges to ensure well maintained, private, and truly accessible bathrooms around their campuses to meet both the rights and needs of students with disabilities. This includes both creating appropriate facilities and committing to maintaining them and ensuring they are used appropriately. Engaging students with disabilities in plans for improving accessibility is important to ensure the bathrooms can best meet the needs for current and future students. Signage may also be helpful to ensure the bathrooms are reserved for those with disabilities. Finally, whenever possible, students with disabilities should be allowed a private bathroom without paying a higher price for the dorm. Improved policy allowing for the provision of support with toileting and other care needs for adults could improve the opportunity for people with disabilities to enter and fully engage in the college experience.

This study is limited by the low number of students with physical disabilities on a bladder and bowel management program. This is a highly selective group of higher education students, making recruitment challenging. However, the goal of this qualitative study was to get an in-depth understanding of participants experiences and perceptions. Additionally, students described overlapping experiences and concerns, reflecting thematic saturation. However, this is also a highly diverse group of students. It is feasible that differences based on factors such as specific bladder or bowel management program, disability type, presence of associated executive functioning limitation or other comorbidities, or mobility aid used were not appreciated. Second, all information about a student's disability and bladder and bowel management was self-reported and no medical records were accessed to verify their reports. Finally, these findings reflect the experiences of those who chose to participate, which may be different from those who elected not to participate.

Next steps include testing the concepts and conceptual model developed in this study in a larger cohort of higher education students with physical disabilities to evaluate the generalizability of the findings. This will further inform clinical practice and the counseling of aspiring students and strengthen advocacy efforts. The relevancy of this model for other applications, such as in employment, could also be tested.

## Conclusions

For college students with physical disabilities, successfully managing toileting needs and formal bladder and bowel programs relies on strong personal skills of the student, especially regarding time management and self-advocacy. For many, these develop or are improved through trial and error during college. Families and medical providers caring for aspiring students should proactively work towards growth in these skills during adolescence. Professors, colleges, and policy often serve as barriers to the student's success. Colleges need to improve the maintenance, privacy, and accessibility of bathrooms and ensure professors are supportive of the accommodation needs of students. Policy is greatly needed to secure caregiver support of adults with disabilities to allow them to fully engage in higher education.

## Data Availability

The datasets presented in this article are not readily available because of ethical and privacy-related concerns due to the potentially identifiable nature of this qualitative data. Requests to access the datasets should be directed to Courtney Streur (coshepar@med.umich.edu).
