# Erratum: “I’m a bathroom expert”: a qualitative study exploring how students with physical disabilities manage bladder and bowel programs during college

**DOI:** 10.3389/fped.2024.1514828

**Published:** 2024-10-28

**Authors:** Frontiers Production Office

**Affiliations:** Frontiers Media SA, Lausanne, Switzerland

**Keywords:** spina bifida, cerebral palsy, disability, persons with disabilities, neurogenic bladder, neurogenic bowel

An Erratum on “I'm a bathroom expert”: a qualitative study exploring how students with physical disabilities manage toileting during college By Okanlami OO, Kreschmer JM, Gupta S, Lee A, Sarma AV and Streur CS (2024). Front. Pediatr. 12:1397229. doi: 10.3389/fped.2024.1397229

Due to an error, the funder for the article processing charge “Dr. Penniston (Department of Urology, University of Wisconsin-Madison)” was erroneously omitted.

The publisher apologizes for this mistake. The original version of this article has been updated.

