# Supplementary material for: “I'm a bathroom expert”: a qualitative study exploring how students with physical disabilities manage toileting during college
Source: Front Pediatr. 2024 Sep 27;12:1397229. doi: 10.3389/fped.2024.1397229 (PMC11466876; doi:10.3389/fped.2024.1397229)
Supplement: Supplementary file 1 [file Datasheet1.pdf]

Thank you for agreeing to participate in this interview. The goal of this study is to learn about your story-- your experience getting to college, what it's been like at college, and your plans and goals for the future. Members from our research team are working to create an educational and inspirational website for adolescents and young adults with physical disabilities. We are hoping to learn from students such as yourself how we can encourage adolescents to think about going to college or pursuing other aspirations after graduating high school. For those interested in college, we would like to learn how to help them plan and prepare for what it may be like. We also hope to use what we learn to share with universities what they are doing well for students with disabilities and what they need to do better. Everything you say will be completely anonymous. Any name you mention during the interview will be removed as it is transcribed. You can skip any questions if you are not comfortable answering them or stop the interview at any point.

Tell me a little bit about yourself. What year are you in college? What is your major or focus of study? What does a typical day look like for you?

> What do you hope to do after graduating?

Tell me about what it was like when you started college.

> Was there anything that was easier than you expected? Or more difficult?

Tell me about the places you have lived and what it has been like getting around while at college.

> Were there any type of accommodations that you requested? How well do you feel the college has provided the accommodations you needed?
>
> Tell me about the things you like about your housing situation. What about any drawbacks to your housing situation?
>
> Have you faced any challenges getting around campus? How has the college supported you in this?

What has it been like interacting with your professors and staff at college? What have they done to support you? What challenges have you faced?

> What do you think your college overall is doing well in supporting people with disabilities?
>
> What do you think they could do better to support people with disabilities?
>
> What has the support from your fellow students been like?

Can you tell me about any activities have you been involved in at college?

Were these activities you also did before college?

Can you talk about what that experience has been like? How has being involved in these activities outside of classwork impacted you or your college experience?

If you haven't been involved in any activities, can you tell me why.

Can you tell me more about your experiences with other students at college? How would you describe those relationships?

How has it been establishing a community? Were there any particular activities, groups, or other ways that you found helpful for making friends or finding a community?

What about ways that made establishing a community challenging?

How does your community at college compare to what you had in high school or at home?

Tell me about any challenges you have faced in developing your community.

Have you had any interest or experience dating while in college?

Do you mind sharing more about this?

How has dating been for you overall? How does your dating life compare to what you would hope for or expected before getting to college?

Our next set of questions are about specific concerns many adolescents have when considering going to college as well as general advice you may have for children and adolescents with physical disabilities.

Often people with physical disabilities require a bladder and bowel program, such as self-cathing or using enemas. Just the thought of having to do a bladder and bowel program while at college can be a huge barrier to even considering going to college for some adolescents. Would you mind sharing if you have a bladder and bowel program?

What has it been like doing these programs while at college?

Do you find you have adequate privacy so you feel comfortable?

Were there any learning curves as you got used to doing it away from home? Or adaptations that you had to make?

Has either your bladder or bowel program changed at all since getting to college?

Do you feel you are more or less strict with staying on your program since going to college? For example, are you extra careful to keep from getting sick? Or are you more relaxed about it?

How common is it for you to skip parts of your program, such as cathing as often as you should or doing your bowel regimen as often as your doctor recommends?

Is there anything about college that makes it harder to stay on a schedule or stay consistent with your program?

Have you found anything that helps you stay on track?

Have you experienced any new issues with your bladder or bowel since being at college (e.g., new leakage, UTIs, accidents)?

How have you managed those?

If you think back to yourself as a middle or high schooler and what you were going through at the time, what do you wish you knew at the time that you know now? What do you wish you could you tell the say 12-year-old version of yourself?

What advice or encouragement do you have for current middle and high schoolers with a physical disability?

We are currently working to build an app or website that helps educate adolescents with physical disabilities about their sexual and reproductive health. We would like to learn from how you like to learn and interact with social media and websites to learn how to get some of the information out.

Say you were to have a question about a sensitive topic such as what sex is like with a physical disability or a specific issue or concern you may be having with sex that you think may be relate to your disability. Where would you go to learn more about it and what can be done?

Have you had any experience trying to get answers about something about your sexual or reproductive health? Where did you go? What was it like? How helpful was the information you found?

Where would you prefer to go to learn about sensitive topics such as the best sexual positions for someone with a disability or what to do about decreased sensation of the genitals?

Say you were in charge of creating some way for other young adults with physical disabilities to learn about sensitive topics related to their sexual and reproductive health. What would you make? For example, would you build a website or app, create some sort of a support group where people could talk together, or use social media?

Do you like to read written materials or watch videos to learn new information?

Do you think using social media like TikTok or Instagram could be helpful?

Is there anything that you feel the University should know about what your experiences have been like? What about other students? In what ways, if any, could other people better support students with disabilities?

When you think about your life up to now, what has been key to your success getting you to this point?

What do you think will be key to your success going forward?
